# Risk Prediction Models for Post-Stroke Dementia

**DOI:** 10.3390/geriatrics2030019

**Published:** 2017-06-22

**Authors:** Eugene Yee Hing Tang, Louise Robinson, Blossom Christa Maree Stephan

**Affiliations:** Newcastle University Institute of Ageing, Newcastle University, Campus for Ageing and Vitality, Newcastle upon Tyne NE4 5PL, UK; e.y.h.tang@newcastle.ac.uk (E.Y.H.T.); a.l.robinson@newcastle.ac.uk (L.R.)

**Keywords:** stroke, dementia, risk prediction

## Abstract

A strong association exists between stroke and dementia with both diseases linked to ageing. Survival rates from stroke are improving which would equate to an ever-expanding population of patients at risk of future dementia. Early or timelier identification of dementia has become a priority in many countries, including the UK. Although screening for dementia is not advocated, targeting at risk populations could be used to reduce an individual’s risk via intervention (i.e., personalised medicine), where available. One approach to improving identification of high-risk dementia individuals is using risk prediction models. Such models could be applied to stroke survivors. Dementia risk prediction models specific to stroke survivors have recently been developed and will be discussed here.

## 1. Introduction

One in three people will experience stroke, dementia or both at some stage in their lives [1,2]. Stroke is known to be a leading cause of morbidity worldwide not only because of its effect on physical function but also because it is associated with an increased risk of cognitive impairment and dementia [3,4,5]. Indeed, stroke is currently the second most common cause of acquired cognitive impairment and dementia [6]. Around one in ten patients develop dementia following their first stroke [4] and the annual incidence rate of dementia is around 3% per year in stroke survivors [7]. Further, the annual dementia conversion rate (7.6%) is higher than stroke recurrence (2.2%) [8]. An increasingly ageing population coupled with the decline in mortality after stroke [9] means that post-stroke dementia (PSD) will become more prevalent particularly since the risk of stroke [10] and dementia [11] rise exponentially with age. 

Impaired cognition following stroke has traditionally been associated with vascular disease. Cerebral small vessel disease is thought to be a major contributor to vascular dementia and is often considered to be a distinct entity from Alzheimer’s disease [12]. However, vascular contributions are now recognised as playing an important role in all-cause dementia and cognitive impairment [13]. To highlight this overlap further, there is evidence that neurodegenerative forms of dementia e.g., Alzheimer’s disease also exist in the context of PSD with estimates of 19% to 61% [14]. The temporal relationship between vascular and neurodegenerative processes and the subsequent development of PSD remains important to clarify.

## 2. Risk Factors for PSD

Numerous risk factors for cognitive decline and dementia have been identified in stroke survivors. These include well-known risk factors for dementia such as age and a history of cardio-metabolic disease, atrial fibrillation, lower educational attainment as well as stroke specific factors (i.e., severity, aphasia, recurrent stroke), pre-stroke cognitive impairment and neuroimaging markers including severity of white matter changes, medial temporal lobe atrophy and hippocampal changes (e.g., volume, mean diffusivity and connectivity) [15,16,17]. A previous systematic review confirmed that older age, low educational attainment, previous cognitive decline, premorbid disability and vascular risk factors (diabetes and atrial fibrillation) were significant predictors of PSD [4]. Recent studies have also suggested that some post-stroke symptoms e.g., leg paralysis [18] could also help predict future cognitive decline. Each could be used in risk models for predicting future post-stroke cognitive decline and dementia.

## 3. Vascular Intervention and Reducing Risk of Cognitive Impairment and Dementia in Stroke Patients

Given the association between vascular factors and risk of dementia, several trials have been undertaken to determine whether dementia risk can be reduced in stroke patients focusing on vascular based interventions. The results have been largely disappointing. One study, The Perindopril Protection against Recurrent Stroke study, found that blood pressure lowering in individuals with a history of stroke or transient ischaemic attack did reduce the risk of dementia and cognitive decline but associated with recurrent stroke [19]. In a study, of first ever stroke patients, compared to standard care a vascular intervention involving information on the importance of changing lifestyle to preserve brain health, optimization of medical treatment, and co-development of treatment plans between the patient and their general practitioner with specific focus on targets for blood pressure, cholesterol and body mass index did not result in reduced risk of cognitive impairment one year post stroke [20]. In another study, despite intensive vascular risk management (e.g., blood pressure control and lipid lowering) in stroke patients, there was no difference in the rates of dementia after two years compared to those receiving guideline treatment [21]. Similarly, a study over 24 months found no benefit of an intervention including intensive management and motivation for compliance with clinical therapy, blood pressure control, lipid/glycemic control, diet, physical activity and cognitive training, versus standard care on the incidence of post-stroke cognitive decline [22]. Taken together, these results suggest that it is likely that an individual’s risk of post-stroke cognitive impairment and dementia includes vascular as well as non-vascular factors and that both types of variables need to be incorporated into dementia prediction models and targeted in intervention strategies. 

## 4. Non-Cognitive Risk Prediction Models

Risk prediction in the context of stroke is not novel. Indeed, risk models have been developed for predicting stroke in whole populations (i.e., the revised Framingham Stroke Risk Profile [23]) as well as in disease specific groups; including the CHA2DS2-VASc score (incorporating: congestive heart failure, hypertension, diabetes, vascular disease, age, gender and previous stroke or transient ischaemic attack) [24] and the ABC-Stroke score (including prior stroke/transient ischaemic attack, troponin and NT-proBNP) [25] for predicting stroke in patients with atrial fibrillation. Further, stroke specific scores have been developed to predict functional as well as health outcomes following stroke. Scores for predicting functional status include: the ASTRAL (Acute Stroke Registry and Analysis of Lausanne) risk score (incorporating: age at stroke onset, baseline National Institutes of Health Stroke Scale score, time from symptom onset to admission, any stroke-related visual field defect, acute blood glucose and decreased level of consciousness [26]) and DRAGON (incorporating: age, dense middle cerebral artery sign, pre-stroke modified Rankin Scale score, glucose, onset to treatment and the National Institutes of Health Stroke Scale score) [27]. Scores for predicting health status include models for: vascular risk [28], falls [29], pneumonia [30] and mortality [31]. Current guidelines recommend that the CHA2DS2-VASc risk score be used to decide on anticoagulation treatment in the context of atrial fibrillation to reduce an individual’s risk of stroke [24]. Similarly the HAS-BLED score is used alongside this to assess the bleeding risk in patients with atrial fibrillation [24]. 

In contrast to the development and use of scores for predicting risk of stroke and health-related outcomes (i.e., pneumonia, physical function and mortality) post-stroke, to date very few models have been developed for the prediction of cognitive impairment and dementia. Cognitive dysfunction post-stroke is both common and severe particularly as early post–stroke cognition has been associated with long-term functional outcomes [32]. Further, patients and caregivers highlight cognition among the top 10 priorities to life post-stroke with clinicians being urged to increase their attention to cognition post-stroke [33,34]. 

## 5. Risk Prediction Models for Cognitive Decline and Dementia

Criteria for Mild Cognitive Impairment (MCI) describe an intermediate stage between normal cognition and dementia, associated with increased dementia risk, namely Alzheimer’s disease [35]. It is thought that this stage could be a treatment target whereby interventions are employed to modify disease progression. However, in stroke patients, criteria for MCI are poor at predicting risk of future dementia [36]. Instead, the term vascular cognitive impairment (VCI) was developed to include all states of cognitive impairment associated with a vascular disorder [13]. Vascular Cognitive Impairment no Dementia (VCI-ND) sits within the spectrum of VCI and is defined as a pre-dementia state associated with increased future risk of cognitive decline and dementia. Although this may be found to be useful for identifying those at greatest risk of future dementia, no single set of criteria for defining VCI-ND yet exist and the predictive accuracy of the state has not been investigated in detail in stroke patients [37]. 

Moving away from the concepts of MCI/VCI, more recently, similar to the development of risk prediction models for other diseases such as cardiovascular disease (CVD) [38,39], acute coronary syndrome [40], diabetes [41], chronic kidney disease [42] and cancer (pancreatic [43], ovarian [44] and breast [45]), new multi-variable models for predicting dementia and more specifically AD have been developed [46,47]. These models (n > 50 models) can be broadly divided into four categories based on their predictor variables including: (1) neuropsychological models; (2) health and vascular risk indices; (3) genetic risk scores; and, (4) multifactorial models typically incorporating demographic, cognitive, health, neuroimaging (MRI) and genetic data. A review of published models found that they have varying predictive accuracy (Area Under the Curve (AUC) range: 0.48 to 0.91) over a follow-up range of one to 20 years. Further, results from external validation analyses were found to be mixed; some models transported well outside the population they were developed in whereas other did not [48,49,50,51,52]. External validation remains important for future models to ensure that they can provide predictions outside of their derivation cohort to ensure real clinical utility [53].

However, it is well established that different disease populations (e.g., people with diabetes, hypertension and stroke), are at greater risk of dementia compared to normal controls. Being able to identify individuals at high risk of dementia co-morbid with other conditions is important as it could: (1) assist in timely identification; (2) allow for personalised treatment to reduce risk, where available; (3) assist with intervention development; and, (4) assist patients, their families and clinicians with future planning. Given this, new disease-specific dementia prediction models have been developed for people with type II diabetes [50] and now also stroke [36,54,55]. 

Table 1 shows each of the different stroke-specific models including their component variables and predictive accuracy. As shown, most models have been used to predict dementia rather than cognitive impairment. Several variables including demographic (i.e., age, education, occupation), cognitive test scores (including global and domain specific function) and neuroimaging markers have been incorporated into the different models and predictive accuracy, measured using the AUC, has been found to be moderate to high (AUC range: 0.83 to 0.85). Only one model has been externally validated with reasonable transportability [54].

Although the primary focus has been on prediction of dementia rather than cognitive impairment, current PSD risk prediction models do not distinguish between dementia subtypes, have not been stratified by age or sex and have been developed predominately in Caucasian samples raising questions of generalisability. Further, most models have a relatively short predictive period and it may be useful to look at the risk of cognitive decline and dementia over longer periods particularly in those who have a stroke at a younger age.

## 6. Conclusions

There has been an increased interest towards timelier identification of those at high risk of dementia so that individuals can access treatment and support earlier. Risk of cognitive impairment and dementia increases after stroke. Therefore, stroke-survivors represent one population where determining future risk of cognitive impairment and dementia could have numerous benefits such as improved access to support, treatment and potentially stricter adherence to secondary vascular prevention to improve prognosis. Further work is required to develop and validate models to predict post-stroke cognitive impairment and dementia and to assess their feasibility, cost-effectiveness and acceptability to patients as well as health care professionals. Although we may develop and validate models in various populations, risk assessment for PSD is not without its challenges. In particular, assessing the acceptability to patients and their families is even more important given the potential psychological effects a “high risk for future dementia” assessment outcome may have. Future studies need to assess how this process could take place in post-stroke clinical care to ensure universal acceptability of such an approach. 

## Figures and Tables

**Table 1:** Models developed for predicting post-stroke cognitive impairment and dementia: Including component variables and predictive accuracy.

Reference and Cohort Used	Sample Size	Follow-up	Outcome	Predictor Variables	Predictive Accuracy	Validation
Kandiah [54] *Tertiary Stroke Clinic*	209	6-m	CI	Age, education, acute cortical infarcts, white matter hyperintensity, chronic lacunes, global cortical atrophy and intracranial large vessel stenosis	AUC = 0.83 (95%CI: 0.77–0.88)	Yes, AUC = 0.78 (95%CI: 0.70–0.85)
Lin [55] *Acute ischaemic stroke patients admitted to neurology department*	283	3-m	Dementia	Age, previous occupation as a laborer, prior stroke, left carotid vascular territory, moderate to severe stroke severity, cognitive impairment, poor functional status at admission	Correct classification = 93.4% of patients	No
Stephan [36] *Population based cohort study*	2640	2-yrs	Dementia	Subjective memory complaint, CAMCOG learning memory and praxis scores	AUC = 0.85 (95%CI: 0.77–0.94)	No

**Key: 95%CI** 95% Confidence Interval; **AUC** Area under the curve; **CAMCOG** Cambridge Cognitive Examination; **CI** Cognitive Impairment; **m** Months; **yr** years.
